# Electrochemical behavior of elemental alloy anodes in solid-state batteries

**DOI:** 10.1021/acsenergylett.4c00915

**Published:** 2024-05-08

**Authors:** Won Joon Jeong, Congcheng Wang, Sun Geun Yoon, Yuhgene Liu, Timothy Chen, Matthew T. McDowell

**Affiliations:** † School of Materials Science and Engineering, Georgia Institute of Technology, Atlanta, Georgia 30332, United States; ‡ George W. Woodruff School of Mechanical Engineering, Georgia Institute of Technology, Atlanta, Georgia 30332, United States

## Abstract

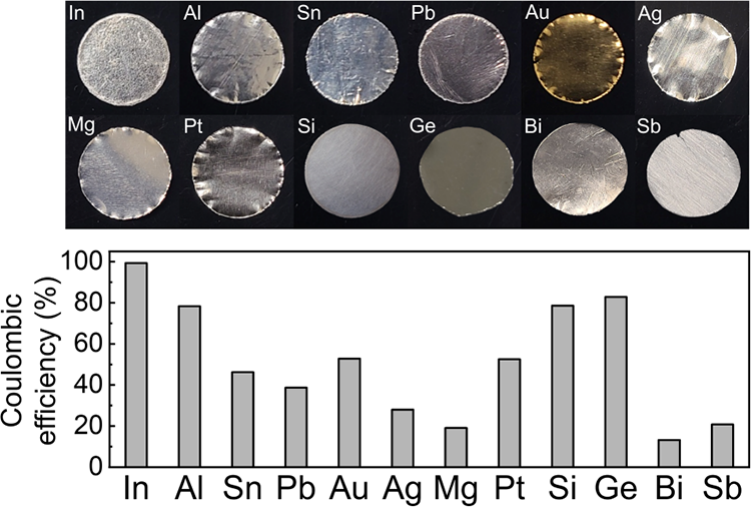

Lithium alloy anodes in the form of dense foils offer significant potential advantages over lithium metal and particulate alloy anodes for solid-state batteries (SSBs). However, the reaction and degradation mechanisms of dense alloy anodes remain largely unexplored. Here, we investigate the electrochemical lithiation/delithiation behavior of 12 elemental alloy anodes in SSBs with Li_6_PS_5_Cl solid-state electrolyte (SSE), enabling direct behavioral comparisons. The materials show highly divergent first-cycle Coulombic efficiency, ranging from 99.3% for indium to ∼20% for antimony. Through microstructural imaging and electrochemical testing, we identify lithium trapping within the foil during delithiation as the principal reason for low Coulombic efficiency in most materials. The exceptional Coulombic efficiency of indium is found to be due to unique delithiation reaction front morphology evolution in which the high-diffusivity LiIn phase remains at the SSE interface. This study links composition to reaction behavior for alloy anodes and thus provides guidance toward better SSBs.

The drive toward widespread vehicle electrification has increased the need for batteries with higher energy and power density, along with improved safety. The solid-state battery (SSB) is a key technology for fulfilling these demands, with its potential for bipolar stacking, utilization of high-capacity electrode materials, and the absence of flammable liquid electrolytes. Development of high-capacity anodes for SSBs is, at present, largely focused on lithium metal or silicon active materials, which is due to their high specific capacity that could enhance the stack-level energy density of SSBs. However, technical challenges related to both electrodes are yet to be fully resolved. Lithium dendrite penetration through the solid-state electrolyte (SSE) can result in short circuits, and void formation at the interface between lithium metal and the SSE increases impedance and causes current constrictions that subsequently give rise to dendrite formation.^1,2^ Particulate silicon anodes have required high uniaxial stack pressure to maintain contact between particles to ensure effective ionic and electronic conductivity.^3−5^ Furthermore, pores or other inactive material within silicon electrodes reduce energy density compared to the theoretical value.

Dense metal foil electrodes that alloy with lithium offer an entirely different anode concept with tantalizing potential performance benefits.^6−8^ Due to their dense architecture and their unique ability to function as both lithium host material and current collector when sufficiently thick, a considerable enhancement in cell energy density can be achieved, rivaling that of silicon-based composites or excess lithium metal electrodes.^9^ Furthermore, the extensive set of electrode fabrication steps associated with conventional slurry casting can be removed by directly incorporating foil anodes. Because of these attributes, foil electrodes have been widely explored in liquid-electrolyte lithium-ion batteries as pure metals or alloys.^6−14^ However, the problem of excessive solid-electrolyte interphase (SEI) growth on the steadily growing internal porosity that forms due to lithiation/delithiation-induced volume changes has prevented long-term cycling stability and stymied research and development progress.^9,10^

Alloy foil anodes have recently been shown to exhibit excellent performance in SSBs because of the solid-state chemo-mechanical environment that is distinct from that of liquid electrolytes. Any internal surface area, such as cracks or pores, created by the volume change of the foils remains unexposed to the SSE, which significantly reduces the extent of SEI formation compared to liquid electrolytes.^3,5,15^ Generally, lithium alloy materials have good chemical compatibility with SSEs due to their higher alloying potentials than lithium metal,^3,6^ which also mitigates dendritic growth of lithium due to the lack of lithium deposition.^16^ Alloy foil anodes in SSBs have been investigated mainly in their prelithiated forms, or with modified microstructure.^11,17−29^ Indium and physically alloyed lithium–indium foils are commonly used as anodes in laboratory SSB cells because of their simple fabrication, constant potential during alloying/dealloying, and stable cycling performance.^11,17−21^ Abundant and cost-effective aluminum foils are particularly attractive beyond indium because of the possibility to attain high specific energy/energy density comparable to that of SSBs with dense silicon anodes.^3^ Recent reports on aluminum-based anodes have shown stable cycling in SSBs by forming Al–In multiphase microstructures and LiAl alloys.^22−24^ Silver and magnesium foils have also been widely employed to form lithium-rich solid solution alloys, which minimizes lithium dendrite growth and contact loss at the SSE/electrode interface.^25−29^ Despite the growing use of alloy foil anodes in SSBs, the reaction mechanisms and degradation behavior of the range of elemental materials that can alloy with lithium are not well understood compared to within lithium-ion batteries with liquid electrolytes.

Here, we investigate the electrochemical alloying and dealloying behavior of a wide array of candidate alloy foil anodes in SSBs using the argyrodite sulfide electrolyte Li_6_PS_5_Cl. Twelve representative alloy materials (indium, aluminum, tin, lead, gold, silver, magnesium, platinum, silicon, germanium, bismuth, and antimony) were selected for investigation. Although silicon, germanium, bismuth, and antimony cannot be processed into thin foils due to their limited ductility, wafers or disk electrodes with excess active materials have shown stable cycling in SSBs.^30^ Substantial differences of Coulombic efficiency (CE) were observed during the first charge/discharge cycle of the various electrodes, with the main cause of inefficiency being lithium trapping within the foil that manifests differently depending on material. The general mechanisms underlying lithium trapping in alloy foil anodes are found to be (1) the formation of the delithiated pure metal with low lithium diffusivity that prevents lithium transport to the SSE interface and (2) contact loss at the SSE interface. Interestingly, different elemental electrodes show substantially different reaction front morphology evolution during delithiation, which directly controls lithium trapping and leads to different initial CEs. By comparing behavior and trends over a wide array of different metal electrodes for SSBs, this work provides important insight into the key factors that enhance and limit performance.

Figure 1 shows the first-cycle galvanostatic voltage curves from 12 different alloy foil anodes in solid-state half cells, all utilizing Li_6_PS_5_Cl (LPSC) SSE and lithium metal counter electrodes. All active materials had purity ≥99.9% (Supporting Information Table S1 shows details). The half cells were tested with a current density of 50 μA cm^–2^ and a voltage range of 0–1.5 V vs Li/Li^+^. This relatively low current density was used to prevent possible short circuiting during lithium plating on the lithium metal counter electrode and to obtain maximal specific capacity. During these tests, the cells were held under a uniaxial stack pressure of 8 MPa at either room temperature (25 °C, black curves) or 60 °C (red curves). Control experiments with lithium symmetric cells tested under identical current density and stack pressure conditions showed flat voltage curves with no polarization (Figure S1a), indicating that lithium behaves as a reliable counter electrode under these conditions. Detailed capacity metrics from these tests are in Table S2.

**Figure 1:**
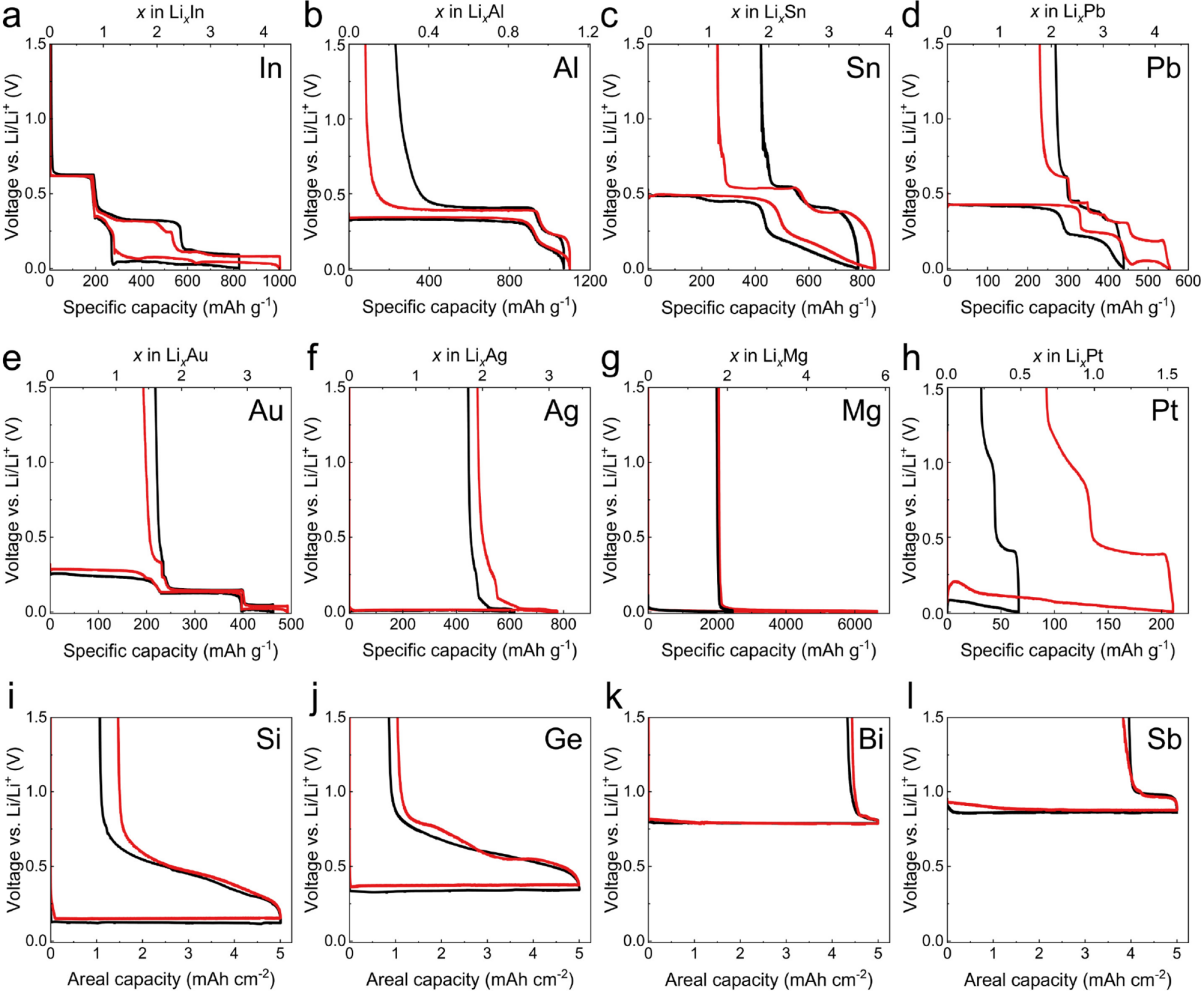
First-cycle galvanostatic voltage curves of dense alloy anodes in solid-state half cells utilizing Li_6_PS_5_Cl SSE and lithium metal counter electrodes. Half cells were tested under a uniaxial stack pressure of 8 MPa and a current density of 50 μA cm^–2^ at both 25 °C (black) and 60 °C (red). (a) Indium, (b) aluminum, (c) tin, (d) lead, (e) gold, (f) silver, (g) magnesium, (h) platinum, (i) silicon, (j) germanium, (k) bismuth, and (l) antimony. The specific capacities and stoichiometric ratios for thick electrodes (silicon, germanium, bismuth, and antimony) were not calculated due to significant excess material in the electrode.

The indium foil working electrode (Figure 1a) exhibited three distinctive lithiation plateaus at 0.63, 0.34, and 0.05 V. The first two plateaus represent the two-phase reactions of indium to form the LiIn and Li_5_In_4_ intermetallic compounds.^21^ After the nucleation overpotential, the extended third plateau at 0.05 V indicates the complete alloying of the indium foil, resulting in the formation of the lithium-rich Li_13_In_3_ phase. Upon delithiation, two plateaus appeared at 0.10 and 0.32 V, which are associated with the dealloying of Li_13_In_3_ into Li_2_In. Subsequently, two overlapping delithiation plateaus to form Li_2_In and Li_3_In_2_ emerged with relatively flat voltage profiles. The coexistence of the Li_2_In and Li_3_In_2_ phases can be advantageous for the delithiation of the Li_3_In_2_ phase, due to the comparatively lower energy barrier for lithium diffusion in the Li_2_In phase and the very high lithium diffusivity of the LiIn phase.^31,32^ The indium foil achieved nearly complete reversibility, exhibiting an extremely high CE of 99.3% at 25 °C and 99.8% at 60 °C. This value is by far the highest of all foils tested in Figure 1, suggesting that indium may exhibit unique reaction pathways.

In contrast to the indium foil, the aluminum foil exhibited a single flat plateau at 0.33 V during lithiation followed by a sloping region at the end of lithiation (Figure 1b). The flat region corresponds to the two-phase reaction of α-Al to form the β-LiAl phase.^10,33^ The sloping portion at the end of lithiation arises from single-phase Li insertion due to the solubility range of the β-Li_*x*_Al phase,^34^ ultimately resulting in a stoichiometry of *x* = 1.108 within this phase. While the aluminum foil showed good reversibility within this solubility range, full reversibility was not attained in the two-phase region, as evidenced by first-cycle CEs of 78.3% at 25 °C and 92.5% at 60 °C. This behavior indicates the incomplete recovery of β-LiAl back to the α-Al phase, implying that Li remains trapped within the β-LiAl phase throughout the foil. The increase of CE when increasing the temperature to 60 °C suggests that diffusion and/or transport limitations contribute to this trapping.

**Figure 2:**
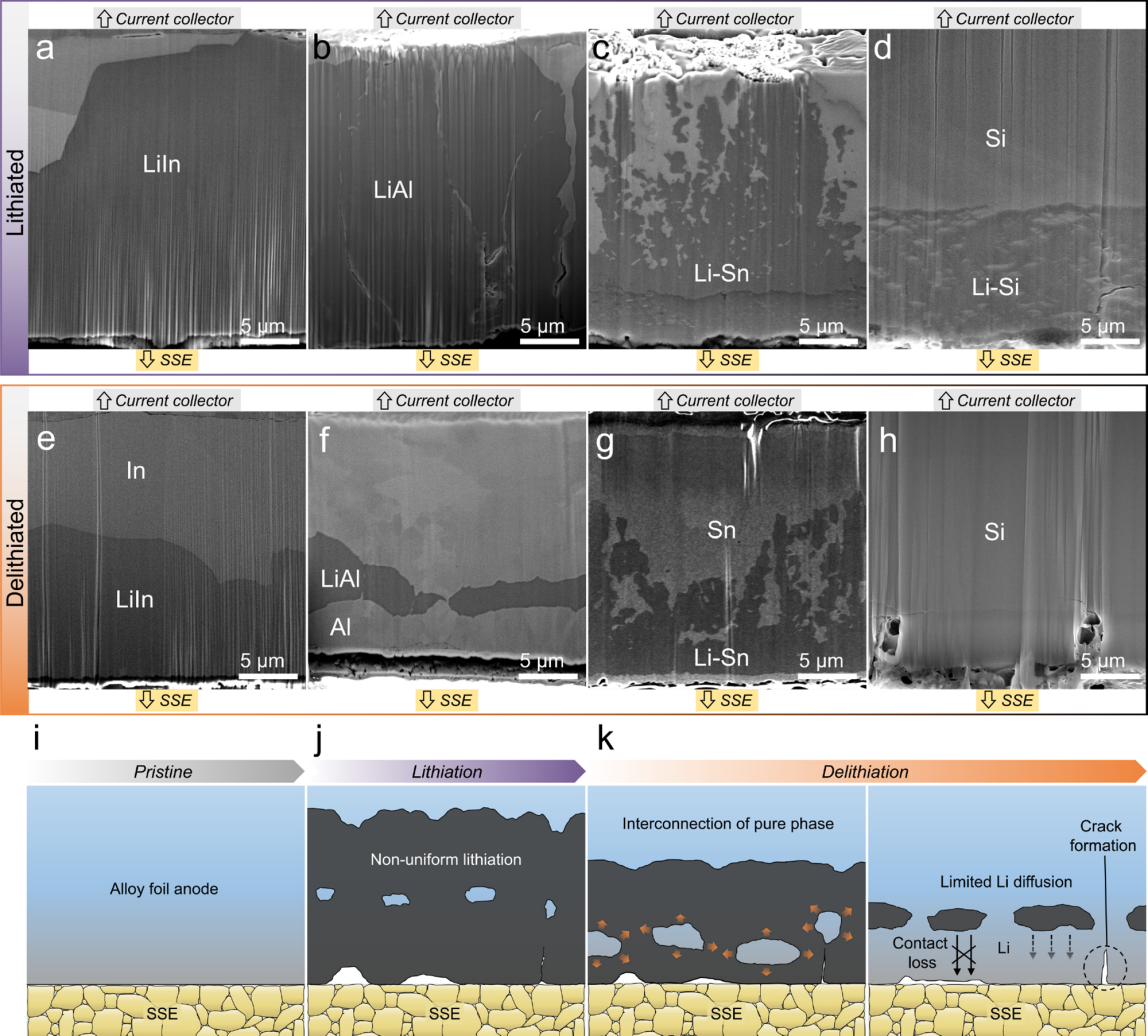
Morphological investigation of reaction progress and lithium trapping within electrode cross-sections. Cross-sectional cryo-FIB-SEM images of dense alloy anodes (a–d) after lithiation to 1.5 mAh cm^–2^ and (e–h) after delithiation: (a, e) indium, (b, f) aluminum, (c, g) tin, and (d, h) silicon. The indium foil was partially delithiated to 1.0 mAh cm^–2^ to ensure LiIn remained. For imaging the morphology and compositional contrast of (de)lithiated foils, a through-the-lens detector (TLD) was used to simultaneously capture secondary electrons (SE) and backscattered electrons (BSE). (i–k) Schematics showing the proposed lithium trapping mechanism in dense anodes that undergo two-phase reactions. (i) Pristine electrode in contact with SSE. (j) Lithiation causes progression of the reaction front into the foil along with unreacted regions within the lithiated material and possible formation of voids and cracks. (k) Delithiation causes two-phase extraction of lithium from the lithium alloy phases, causing phase front movement and growth of the elemental phase, which eventually causes lithium trapping behind the pure phase as well as exacerbated contact loss.

The tin, lead, and gold foils transitioned through a range of intermetallic compounds via two-phase reactions during lithiation, as depicted in Figure 1c–e. Notably, the gold electrode featured very low overpotentials during transitions between Li–Au compounds (i.e., *x* > 1.5 in Li_*x*_Au), indicating facile transformation kinetics. However, these foils showed poor reversibility (CE values of 46.2% for tin, 38.7% for lead, and 52.8% for gold), characterized by extreme irreversibility of the final two-phase dealloying reaction, which involves removal of lithium to form the elemental material.^35−38^ This finding is likely because the elemental metallic phases generally feature much lower Li diffusivity than the alloyed phases.^39−42^ The slight improvement of reversibility at the increased temperature of 60 °C can be attributed to the enhanced kinetics of solid-state lithium diffusion within the various phases, but there are still diffusion constraints.

Silver and magnesium foil electrodes (Figure 1f,g) showed lithiation with subtly sloping voltage curves close to 0 V vs Li/Li^+^, along with high specific capacities. These two elements are recognized to react with lithium through single-phase solid solution reactions with electrode potentials near that of lithium metal, as observed in Figure 1f,g. Due to the unlimited solubility of lithium within the lithium-rich Li–Mg solid solution (β-phase) after transitioning from the α-phase,^43^ the magnesium foil exhibited the very high discharge specific capacity of 6647 mAh g^–1^ at 60 °C (Figure 1g). Additionally, the relatively high lithium diffusivity within the β-phase (∼10^–9^ cm^2^ s^–1^ at 50 °C) and contact retention at the SSE interface may contribute to this high specific capacity.^27,28^ However, both foils featured poor CE even at elevated temperatures (CE values of 38.3% for silver and 69.2% for magnesium at 60 °C), as evidenced by the sharp voltage polarization approximately at *x* = 2 in both Li_*x*_Ag and Li_*x*_Mg. Platinum (Figure 1h) also showed a sloping profile at relatively low potentials, but with much lower specific capacities along with poor reversibility.

Thick wafers of silicon (500 μm) and germanium (400 μm) tested as working electrodes displayed flat lithiation plateaus (0.12 V for silicon and 0.34 V for germanium) until arbitrary cutoffs of 5 mAh cm^–2^ (Figure 1i,j) were reached in both cases. These flat voltage profiles are associated with the lithiation of crystalline silicon and germanium to form amorphous lithiated phases.^44,45^ The current was reversed after reaching these areal capacity values for both cells. CEs greater than 78% were achieved at 25 °C in both cases, which are high values given the relatively large areal capacity utilized. The single-phase delithiation behavior of Li_*x*_Si and Li_*x*_Ge may contribute to this reversibility, since the absence of a sharp reaction front and the presence of lithium concentration gradients may reduce the isolation of lithium-rich alloy phases. Finally, Figure 1k,l show the voltage profiles of cells with bismuth and antimony working electrodes. Both showed flat two-phase lithiation profiles at the relatively high electrode potentials at which these materials operate (0.79 V for bismuth and 0.86 V for antimony). Furthermore, both showed very low reversibility upon Li removal (CE values of 13.3% for bismuth and 20.8% for antimony). This may be due to the two-phase reaction upon delithiation, which is different than the silicon and germanium cases.

The data from different materials tested under identical conditions in Figure 1 allows for instructive comparisons to be made. All elemental materials except for platinum were readily lithiated in the solid-state cells to form either terminally lithiated compounds (with thin foils) or to reach relatively high areal capacity cutoffs (with thicker samples). This result demonstrates that these materials can be lithiated without liquid electrolyte, relying on solid-state diffusion within the active materials. Increasing the operating temperature from 25 to 60 °C increased the attained capacity during discharge and the Coulombic efficiency for most materials, indicating more facile diffusion and phase transformations at elevated temperature. Additionally, all materials in Figure 1 that showed final two-phase delithiation to the elemental phase exhibited relatively poor CE, except for indium. As previously mentioned, this result generally suggests that the two-phase formation of the fully delithiated (elemental) phase promotes lithium trapping, but the unique behavior of indium motivates further investigation.

The differences between the electrochemical reversibility of indium and other materials that exhibit two-phase delithiation suggest different reaction pathways. To visualize the distribution of lithium alloy phases within the electrodes, we used cryogenic focused-ion-beam scanning electron microscopy (cryo-FIB-SEM). Ion milling was carried out at cryogenic temperature (−140 °C) to prevent possible beam damage. Indium, aluminum, and tin were selected as they undergo two-phase delithiation but show different CEs, and silicon was chosen for comparison due to its single-phase delithiation characteristics. Figure 2a–d show indium, aluminum, tin, and silicon electrodes after lithiation to a capacity of 1.5 mAh cm^–2^, which is less than the full theoretical capacity. Figure 2e–h show similar foils but after lithiation to 1.5 mAh cm^–2^ and then delithiation to a 1.5 V cutoff or a 1.0 mAh cm^–2^ capacity cutoff in the indium case (see Figure S2 for voltage curves). These images show clear contrast between the lithiated regions (darker) and the unlithiated or delithiated regions (lighter), which results from the different average atomic number between regions that contain lithium and those that do not.

Figure 2a shows an indium foil after (partial) lithiation. The flat plateau at 0.63 V (Figure S2a) corresponds to the two-phase reaction of indium to form LiIn. The LiIn phase, characterized by the dense region with darker contrast, extends from the SSE below the image, with the unlithiated indium remaining at the top near the current collector. This indicates that LiIn nucleates and grows at the SSE/indium interface. After partial delithiation (Figure 2e), the LiIn phase covers less of the foil but has disappeared from the current collector side and remains in contact with the SSE interface. This suggests that the two-phase reaction front moves toward the SSE interface from the back of the foil (i.e., the current collector side) during delithiation of LiIn, with the LiIn remaining in continuous contact with the SSE and enabling delithiation without trapping. The exceedingly high diffusion coefficient of lithium within LiIn (∼10^–6^ cm^2^ s^–1^)^32^ seemingly promotes transport of lithium to the SSE through the LiIn phase while this phase shrinks. This behavior was further examined after the complete lithiation of indium foil to LiIn (lithiation to 0.34 V, see Figure S3 for cryo-FIB-SEM images and Figure S4 for voltage curves). The homogeneous LiIn phase observed in Figure S3a confirms the complete reaction of indium. After partial delithiation (Figure S3b), the LiIn phase was still in contact with the SSE, again enabling transport to the SSE interface. This suggests that the nucleation of the indium phase at the current collector (instead of near the SSE) is likely due to transport effects and possibly concentration gradients within the LiIn phase.

Figure 2b shows an aluminum foil electrode after partial lithiation; the foil mostly contains the β-LiAl phase with narrow interspersed regions of unlithiated α-Al. These α-Al regions, which may form at grain boundaries of β-LiAl,^46^ also feature cracks and internal pores. In the delithiated aluminum foil (Figure 2f), isolated β-LiAl regions are visible that are separated from the SSE interface by a layer of delithiated α-Al with a thickness of ∼3 μm. This observation is a direct demonstration of lithium trapping, as it appears that the lithium within the β-LiAl cannot be transported through the delithiated α-Al to reach the SSE interface. The pure aluminum exhibits a low lithium diffusion coefficient and thus cannot sustain transport of lithium to the interface, resulting in voltage polarization before all lithium is removed. The cryo-FIB-SEM image of a tin foil after lithiation (Figure 2c) reveals a distinctive morphological distribution of the Li–Sn phase. The scattered nature of the lithiated region suggests nonuniform movement of the reaction front during lithiation, which would concentrate stresses that arise due to volume expansion. After partial delithiation of a different tin sample (Figure 2g), the distributed reacted region is again visible. A distinctive delithiated tin layer with lighter contrast is also visible at the interface with the SSE, similar to the aluminum but much thinner. We hypothesize that this delithiated tin region causes trapping that contributes to the very low CE observed in the electrochemical data in Figure 1c. Finally, the partially lithiated silicon electrode shows a lithiated layer with a thickness of ∼10 μm. In this layer, the darker Li_*x*_Si phase surrounds included regions of lighter silicon that are likely unreacted and still in the crystalline phase. After delithiation of the silicon electrode (Figure 2h), there are no obvious regions of trapped lithium, but trapped lithium is likely harder to detect due to the single-phase reaction during silicon delithiation and associated lack of sharp reaction fronts.^47^

Considering the imaging data in Figure 2, the main difference between indium and the other materials is the continued presence of the LiIn phase at the SSE interface during delithiation, in contrast to the growth of the pure elemental materials (aluminum and tin) near the SSE interface during delithiation to cause lithium trapping. Pure indium begins to form at the current interface with the LiIn phase maintaining contact with the SSE interface, whereas the pure aluminum and tin grow at the SSE interface. There is thus a clear mechanistic distinction among these materials during two-phase dealloying. This behavior is likely the origin of the very high CE for indium foils shown in Figure 1a, while the other materials exhibit lower CEs. The transport properties and distribution of the phases within the material itself are therefore both important for determining reversibility. In addition to this trapping effect, it is possible that contact loss at the SSE/electrode interface could arise during local volume contraction associated with delithiation, which would manifest in similar ways as contact loss during lithium stripping.^48−50^ While this may play a role in our experiments, it is difficult to determine with *ex situ* cryo-FIB since the material stack is removed from the cell (and the applied stack pressure) for imaging. The schematics in Figure 2i–k show the process for lithiation and delithiation of two-phase materials other than indium, where delithiation causes growth of the pure phase near the SSE interface which induces trapping of lithium.

Further insight into reaction mechanisms in solid-state systems can be gleaned by comparing to the electrochemical behavior of elemental alloy anodes in cells with liquid electrolyte. Figure 3 shows the first-cycle galvanostatic voltage curves of nine elemental anodes with liquid electrolyte composed of 1.0 M LiPF_6_ in ethylene carbonate/dimethyl carbonate (EC/DEC; 1:1 by volume) with 10 vol % fluoroethylene carbonate (FEC). For direct comparison, the liquid-electrolyte-filled pouch-type half cells were subjected to the same uniaxial stack pressure (8 MPa) as used in solid-state half cell tests. Because of the risk of lithium metal permeating the separator under this relatively high stack pressure, a Li_0.5_In alloy counter electrode was employed. Li_0.5_In symmetric cells with liquid electrolyte tested under the same conditions showed low overpotential and constant voltage (Figure S1b), proving that Li_0.5_In functions as a reliable counter electrode.

**Figure 3:**
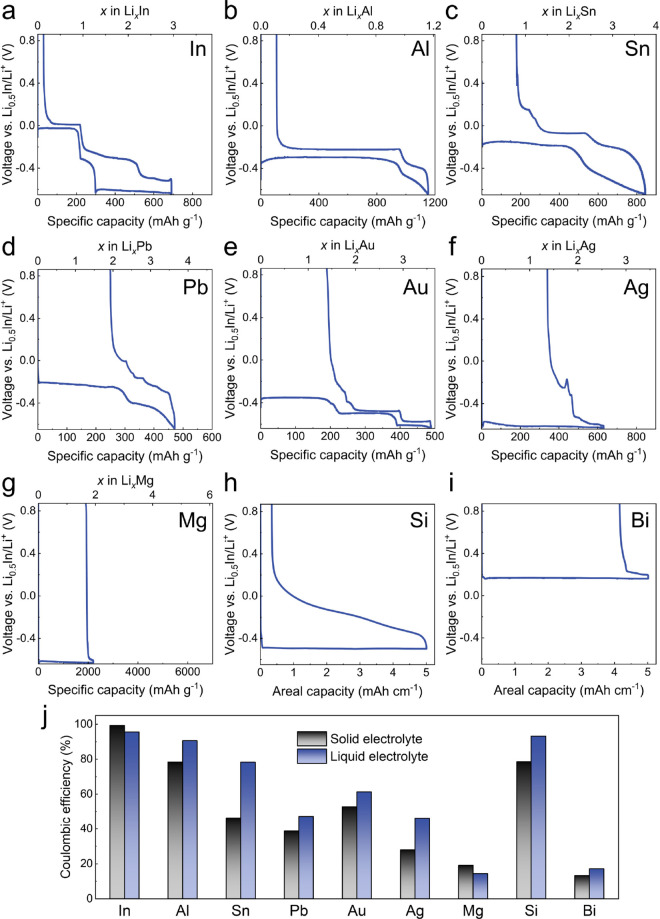
First-cycle galvanostatic voltage curves of dense alloy anodes in liquid-electrolyte pouch cells, utilizing 1.0 M LiPF_6_ in EC/DEC with 10 vol % FEC liquid electrolyte and Li_0.5_In counter electrodes. (a–i) Voltage curves of nine alloy anodes: (a) indium, (b) aluminum, (c) tin, (d) lead, (e) gold, (f) silver, (g) magnesium, (h) silicon, and (i) bismuth. The half cells were tested under 8 MPa stack pressure and 50 μA cm^–2^ at 25 °C within the voltage range −0.63 to +0.87 V vs Li_0.5_In/Li^+^. (j) First-cycle CE comparison of alloy anodes in liquid-electrolyte and SSE cells at 25 °C with stack pressure of 8 MPa.

The voltage curves in Figure 3a–i display similar shapes as those observed in solid-state half cell tests (Figure 1), indicating that the same phase transformations occur. The first-cycle CE values of the alloy electrodes in the liquid electrolyte are notably higher than those in SSB cells (Figure 3j). This increase is caused by liquid infiltration into the cracks and pores that develop in the foil during (de)lithiation, which increases contact area, shortens diffusion path lengths, and limits lithium tripping, thereby enhancing the kinetics of lithium removal and leading to enhanced reversibility of the alloy foil anodes in liquid electrolyte. However, this factor also causes relatively rapid failure of foils in liquid electrolytes, as the increased interfacial area accelerates SEI formation.^9^

The electrochemical behavior of different elemental alloy anodes was evaluated in SSB full cells with LiNi_0.6_Mn_0.2_Co_0.2_O_2_ (NMC622) composite cathodes. These cells were tested under the same stack pressure as the solid-state half cells (8 MPa). To match the cathode loading of 5.0 mAh cm^–2^, a thinner tin foil (10 μm) was used, whereas the thickness of all other electrodes was the same as the half cell tests. The first-cycle CEs of the full cells were in line with the reversibility of the different alloy foil anodes in half cells, as shown in Figure 4a–d (see Table S3 for detailed capacity metrics). The indium foil exhibited the highest first-cycle CE (85.4%) and stable cycling at high capacity (3.5 mAh cm^–2^) for 100 cycles (Figure 4a,e), consistent with its behavior in half cells and the unique reaction front evolution (Figure 2). The lower CE value here compared to the indium half cell likely arises from irreversibility of the cathode. The aluminum and silicon experienced capacity decay and large fluctuations in CE during the initial cycles (Figure 4b,d–f), suggesting continued lithium trapping/recovery and possible contact loss. The relatively stable cycling observed for silicon over aluminum is likely due to homogeneous (de)lithiation of amorphous Li_*x*_Si causing less mechanical degradation, compared to nonuniform reaction in aluminum leading to porosity formation.^12,22,46,47^ The cell with tin showed limited capacity utilization due to dealloying constraints during the final two-phase reaction (Figure 4c,e), although the capacity was relatively stable with cycling. Prelithiation may be a useful strategy to enhance capacity for this material. The average CE values over cycles 2–100 were 99.67% for indium, 98.84% for aluminum, 99.52% for tin, and 100.34% for silicon. Overall, the full cell results are consistent with the behavior of the foils in half cells.

**Figure 4:**
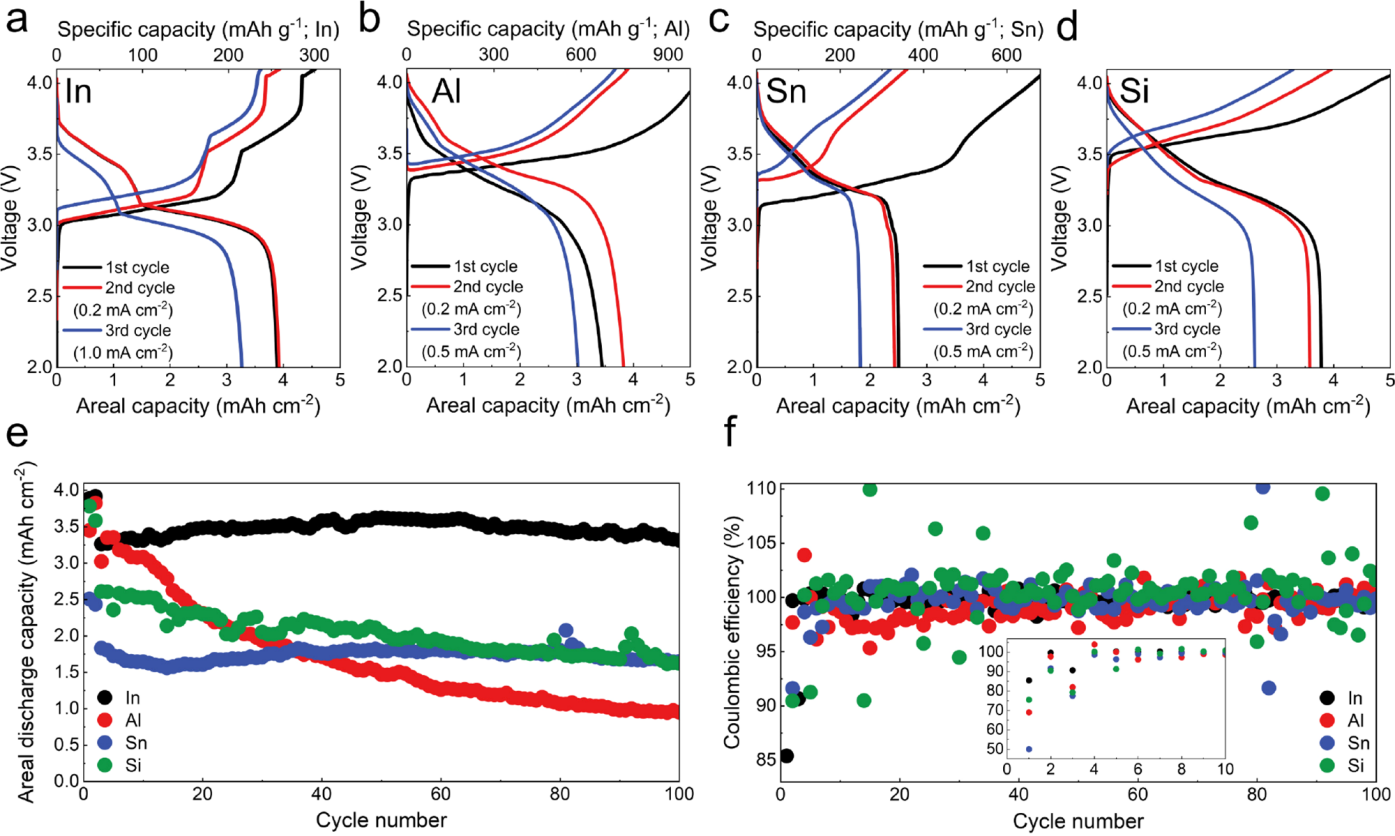
Galvanostatic cycling of dense alloy anodes in solid-state full cells with LPSC SSE and NMC622 cathodes. (a–d) First, second, and third galvanostatic voltage curves from (a) indium (20 μm), (b) aluminum (20 μm), (c) tin (10 μm), and (d) silicon (500 μm) cells. The cathode loading was 5 mAh cm^–2^ (corresponding to a mass loading of 27 mg cm^–2^ NMC622), and the N:P ratios of these cells were 1.02 for indium, 1.07 for aluminum, and 1.15 for tin (calculated based on the Li_3_In_2_, β-LiAl, and Li_7_Sn_2_ phases). (e) Discharge capacities and (f) CE values during galvanostatic cycling of alloy foil anodes. The inset in (f) shows the CE values over the first 10 cycles. A current density of 0.2 mA cm^–2^ was used for the first two cycles followed by 0.5 mA cm^–2^ (except for 1.0 mA cm^–2^ for indium) for the following cycles. The areal charge capacity was limited to 5 mAh cm^–2^ for every cycle. All cells were tested at 25 °C between the voltage range 2.0–4.1 V with a uniaxial stack pressure of 8 MPa.

The rate-dependent electrochemical behavior of selected alloy foil anodes (indium, aluminum, tin, and silicon) was further investigated with cyclic voltammetry (CV, Figure S5) and galvanostatic cycling at different current densities (Figure S6). The CV tests of indium, aluminum, and silicon each showed relatively high reduction peak current densities (1.04 mA cm^–2^ for indium, 0.91 mA cm^–2^ for aluminum, and 1.24 mA cm^–2^ for silicon), indicating fast lithiation kinetics. Indium also exhibited a peak current density on oxidation (1.27 mA cm^–2^) that was as high as the reduction peak current (Figure S5a), suggesting fast alloying and dealloying kinetics. This is distinct from the aluminum cell, which despite similar two-phase delithiation showed lower peak current density (0.419 mA cm^–2^) and thus slower dealloying kinetics. Consistent with these findings, the solid-state full cell with an indium anode demonstrated the highest capacity at higher current densities, while silicon showed the lowest capacity upon increasing the current density to 2.0 mA cm^–2^ (Figure S6). Aluminum exhibited better rate performance compared to tin and silicon. Overall, the good rate performance of indium is consistent with the microstructural evolution observed in Figure 2.

The improved understanding of various alloy materials herein motivates further work to improve battery performance. We find that degradation due to lithium trapping within many alloy anodes occurs even at low current densities and relatively high stack pressures, this suggests that tailoring composition and microstructure of dense alloy foils could be useful to mitigate lithium trapping and enable SSB operation under lower stack pressures. Furthermore, our work points to additional directions for future investigation, including (i) understanding the (electro)chemical stability of SSEs in contact with alloys exhibiting various electrode potentials and (ii) investigating how the changing mechanical properties of alloys during lithiation/delithiation influence contact loss and stress evolution under low stack pressures.

## Supporting Information Available

Materials and methods; voltage curves of symmetric cells; electrode thickness and detailed capacity metrics for solid-state half cell tests; voltage curves for cryo-FIB-SEM samples; additional cryo-FIB-SEM images of indium after lithiation and delithiation; voltage curve for cryo-FIB-SEM samples of indium; electrode thickness and detailed capacity metrics for solid-state full cell cycling tests; cyclic voltammetry curves of solid-state half cells; rate-dependent galvanostatic testing of solid-state full cells (PDF)
