# Supplementary material for: Electrochemical behavior of elemental alloy anodes in solid-state batteries
Source: ACS Energy Lett. 2024 May 8;9(6):2554–63. doi: 10.1021/acsenergylett.4c00915 (PMC11187630; doi:10.1021/acsenergylett.4c00915)
Supplement: Supplementary file 1 — nz4c00915_si_001.pdf [file nz4c00915_si_001.pdf]

Supporting Information

Electrochemical behavior of elemental alloy anodes in solid-state batteries

Won Joon Jeong[1], Congcheng Wang[2], Sun Geun Yoon[2], Yuhgene Liu[1], Timothy Chen[2], Matthew T. McDowell[1,2*]

[1]School of Materials Science and Engineering, Georgia Institute of Technology, Atlanta, GA, 30332, USA

[2]George W. Woodruff School of Mechanical Engineering, Georgia Institute of Technology, Atlanta, GA, 30332, USA.

[*]Corresponding Author: mattmcdowell@gatech.edu

Materials and Methods

Electrode preparation: Indium, aluminum, tin, lead, gold, silver, magnesium, and platinum foils were fabricated by cold-rolling ingots or foils. The rolling was performed unidirectionally using an electric Durston rolling mill equipped with 60 mm diameter rollers to achieve the required foil thickness. Following the rolling process, the foils were polished using abrasive silicon carbide (SiC) paper to remove surface oxide layers before electrochemical testing. Silicon and germanium wafers, bismuth disks, and rectangular antimony pieces, which were made from melting small antimony pieces, were first diced into areas of ~ 15 × 15 mm^2. Subsequently, these pieces were polished with abrasive SiC paper until their diameter and thickness were reduced to the desired dimensions. Materials sourced from multiple vendors are listed in Table S1.

Table S1. Materials used in this study.

Material	Purity	Vendor
In pellets	>99.99%	Kurt J. Lesker
Al foil	>99.999%	Laurand Associates
Sn pellets	>99.99%	Kurt J. Lesker
Pb shot	>99.999%	Kurt J. Lesker
Au foil	>99.99%	Sigma Aldrich
Ag foil	>99.9%	Sigma Aldrich
Mg substrate	>99.9%	MTI
Pt foil	>99.9%	Sigma Aldrich
<110> Si wafers	-	MTI
<100> Ge wafers	-	MTI
Bi foil	>99.999%	GoodFellow
Sb pieces	>99.999%	Kurt J. Lesker

$Li_{0.5}In$ counter electrodes were prepared with lithium (MSE Supplies) and indium foils with an atomic ratio of 1:2 (lithium:indium). These prepared lithium and indium foils were stacked in an indium/lithium/indium configuration and mixed via an accumulative roll bonding process in an Ar-filled glove box. The prepared $Li_{0.5}In$ counter electrodes were punched into disks of 15 mm diameter before electrochemical testing.

Cathode composite electrodes were prepared by blending $LiNb_{0.5}Ta_{0.5}O_3$ (LNTO)-coated single-crystal $LiNi_{0.6}Mn_{0.2}Co_{0.2}O_2$ (NMC622, MSE Supplies),[S1] Li_6PS_5Cl (LPSC, MSE Supplies, Ampcera), and vapor-grown carbon fiber (VGCF, Sigma Aldrich) with a weight ratio of 70:27.5:2.5. LNTO was synthesized by dissolving stoichiometric amounts of niobium ethoxide (Sigma Aldrich, 99.95%), tantalum butoxide (Sigma Aldrich, 99.99%), and lithium acetate (Sigma Aldrich, 99.95%) in dry ethanol (Sigma Aldrich, 99.5%) and stirring for 12 h. NMC622 powder was then added to this solution and mixed via sonication (Branson 1510 Ultrasonic Cleaner) at 40 kHz for 2 h. After sonication, the solvent was evaporated in a vacuum oven, followed by annealing at 450 °C for 1 min. The mixture of LNTO-coated NMC622, LPSC, and VGCF was then added into ZrO_2 jar with eight ZrO_2 balls and sealed in an Ar-filled glove box. The mixture was dry ball milled (Fritsch Pulverisette 7) at a milling speed of 150 rpm for 15 min, repeated three times.

Cell assembly: Solid-state half cells were assembled using elemental working electrodes, LPSC solid-state electrolyte (SSE), and a lithium counter electrode. Elemental electrodes, with a diameter of 10 mm, were prepared through punching or polishing. 90 mg of the ultrafine LPSC powder (particle size ~1 μm) was poured into a polyether ether ketone (PEEK) die (inner diameter: 10 mm) and uniaxially pressed to a pressure of ~250 MPa for 5 min using titanium plungers. Subsequently, the alloy working electrode was added and subjected to further pressing up to ~375 MPa. The lithium counter electrode was attached to a titanium plunger and inserted to contact the densified LPSC pellet. Finally, the alloy anode/LPSC/lithium stack was then uniaxially pressed to ~60 MPa to form interfacial contact between the lithium counter electrode and the LPSC pellet.

Liquid-electrolyte pouch cells were assembled using elemental working electrodes, liquid electrolyte, separators, and $Li_{0.5}In$ counter electrodes. The elemental electrodes were punched or polished into discs with a diameter of 15 mm. The liquid electrolyte used was 1.0 M $LiPF_6$ in ethylene carbonate/dimethyl carbonate (EC/DEC, 1:1 by volume, Sigma Aldrich) with 10 vol% fluoroethylene carbonate (FEC, Sigma Aldrich). Glass fiber membranes (Cytiva Whatman™) were used as separators. The separator was soaked in electrolyte before assembly and an additional 50 μL of electrolyte was added to each cell before vacuum sealing.

The lithium symmetric cell was assembled by uniaxially pressing 90 mg of ultrafine LPSC powder at ~375 MPa for 5 min using titanium plungers. Subsequently, the lithium electrodes were attached to titanium

plungers and inserted to contact both sides of the densified LPSC pellet. The mass of the lithium electrodes was controlled to be the same. Finally, the lithium/LPSC/lithium stack was uniaxially pressed to 60 MPa to form interfacial contact between the lithium counter electrode and LPSC pellet. The $Li_{0.5}In$ symmetric cell was assembled with $Li_{0.5}In$ electrodes, liquid electrolyte composed of 1.0 M $LiPF_6$ in EC/DEC with 10 vol% FEC, and glass fiber membranes as separators. The mass of $Li_{0.5}In$ at each electrode was controlled to be the same.

Solid-state full cells were assembled by first uniaxially pressing 90 mg of ultrafine LPSC powder at ~125 MPa in the PEEK die. Then, 30 mg of cathode composite powder, equivalent to ~5.0 mAh cm^{-2}, was added on top of LPSC pellet and pressed to ~250 MPa. Finally, the alloy electrode was added on the other side of the LPSC pellet and pressed to ~375 MPa for 5 min. All cells were assembled inside the Ar-filled glove box.

Electrochemical measurements: Before electrochemical testing, all cells (solid-state half cells, liquid-electrolyte pouch cells, and solid-state full cells) were sandwiched between steel stack plates, and a constant stack pressure of 8 MPa was applied by tightening nuts at each corner using a digital torque wrench.[S2] Springs at each corner were used to ensure uniform stack pressure distribution across the cell.[S3] The stack pressure was calibrated with a pressure sensor prior to the tests. Solid-state half cell tests were performed with Arbin and Landt Instruments battery cyclers, with a current density of 50 µA cm^{-2} and a voltage range of 0 to 1.5 V vs. Li/Li^+. For high-temperature half cell testing at 60 °C, heat tape was wrapped around the PEEK die during the test. Due to the insulating properties of PEEK, the outer diameter of the die was reduced to ~12 mm to improve heat transfer to the alloy anode/LPSC/lithium stack. The liquid-electrolyte pouch cell was tested with a Neware battery testing system, with a current density of 50 µA cm^{-2} and a voltage range of -0.63 to 0.87 V vs. $Li_{0.5}In$/Li^+. The symmetric cells were cycled for one cycle at a current density of 50 µA cm^{-2} with capacity cutoff of 5 mAh cm^{-2} for each half-cycle. For the solid-state full cell cycling test, a current density of 0.2 mA cm^{-2} was used for the first two cycles and 0.5 mA cm^{-2} for the subsequent cycles, with a voltage range of 2.0 to 4.1 V. The areal charge capacity was limited to 5 mAh cm^{-2} for every cycle. The cyclic voltammetry (CV) tests of solid-state half cells were performed with a Bio-Logic SP-200 potentiostat. The sweep rate was 0.01 mV s^{-1} with a voltage range of 0 to 1.0 V. For the rate performance tests of solid-state full cells, a current density of 0.2 mA cm^{-2} was used for the first two cycles followed by increasing the current density in steps of 0.5, 1.0, and 2.0 mA cm^{-2}, with three cycles for each current density. The current density was then reduced back to 0.2 mA cm^{-2} for the rest of the cycles. All the solid-state cells were tested in Ar-filled glove box. Except for the elevated temperature solid-state half cell tests, all cells were tested at room temperature (25 °C).

Material characterization: Cryogenic focused-ion beam scanning electron microscopy (cryo-FIB-SEM) imaging was carried out using a Thermo-Fisher Helios 5CX FIB-SEM equipped with a Ga^+ source and a Quorum cryogenic stage system. The (de)lithiated electrodes were extracted from the PEEK die inside the Ar-filled glove box and transferred into the SEM chamber, with a few seconds of air exposure. All samples were cooled down to -140 °C prior to FIB milling and imaging to reduce detrimental interactions with the ion beam.[S4] An initial cross-sectional milling utilized 100 nA of beam current with 30 kV accelerating voltage. For final polishing of the cross-section, 2.8 nA of beam current was applied. For imaging the morphology and compositional contrast of (de)lithiated foils, a through-the-lens detector (TLD) was used to simultaneously capture secondary electrons (SE) and backscattered electrons (BSE).

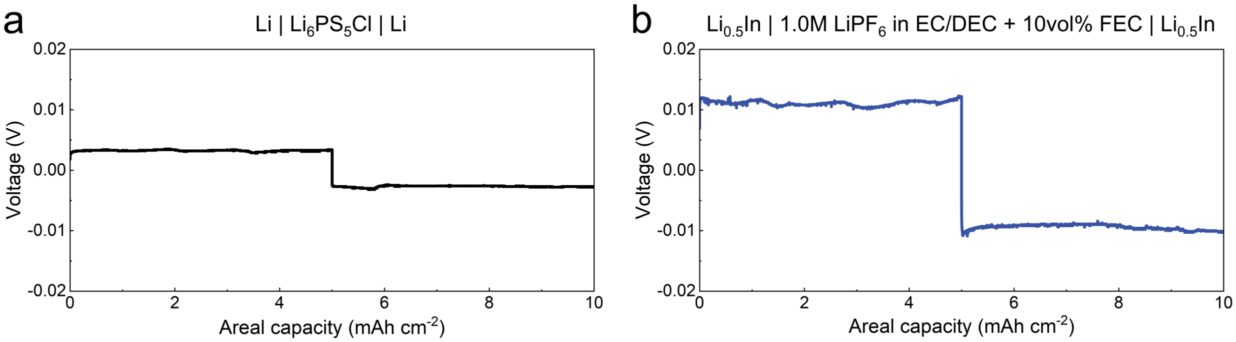

Figure S1. Galvanostatic voltage curves of (a) a lithium symmetric cell with LPSC SSE and (b) a $Li_{0.5}In$ symmetric cell with liquid electrolyte consisting of 1.0 M $LiPF_6$ in EC/DEC with 10 vol% FEC. The symmetric cells were cycled for one cycle at a current density of 50 μA cm^{-2} with a half-cycle areal capacity of 5 mAh cm^{-2}. The cells were tested at 25 °C with a uniaxial stack pressure of 8 MPa. Both cells exhibit flat voltage profiles with minimal voltage and no polarization, showing that these electrodes can effectively be used as counter electrodes in half cells under these conditions.

Table S2. Alloy anode thickness used in solid-state half cell tests, as well as areal discharge/charge capacities, specific discharge/charge capacities, and Coulombic efficiencies values from Figure 1.

Foil anode	Thickness (μm)	Temperature	Areal discharge capacity (mAh cm^{-2})	Areal charge capacity (mAh cm^{-2})	Specific discharge capacity (mAh g^{-1})	Specific charge capacity (mAh g^{-1})	Coulombic efficiency (%)
In	20	60 °C	12.40	12.38	1004.4	1002.4	99.8
		25 °C	10.51	10.44	825.9	820.0	99.3
Al	20	60 °C	5.87	5.44	1101.0	1018.9	92.5
		25 °C	5.11	4.00	1070.6	838.6	78.3
Sn	20	60 °C	11.56	8.02	848.3	588.9	69.4
		25 °C	10.96	5.06	785.8	363.0	46.2
Pb	10	60 °C	5.23	3.06	554.5	324.5	58.5
		25 °C	4.15	1.60	439.7	170.1	38.7
Au	5	60 °C	5.79	3.52	492.5	299.1	60.7
		25 °C	3.39	1.79	462.5	244.2	52.8
Ag	5	60 °C	5.50	2.11	777.0	297.8	38.3
		25 °C	4.37	1.22	616.9	172.5	28.0
Mg	10	60 °C	9.39	6.50	6647.0	4597.4	69.2
		25 °C	2.57	0.49	2458.4	468.7	19.1
Pt	10	60 °C	2.72	1.53	210.9	118.4	56.1
		25 °C	0.88	0.46	66.7	35.0	52.5
Si	500	60 °C	5.0	3.54	-	-	70.6
		25 °C	5.0	3.94	-	-	78.6
Ge	400	60 °C	5.0	3.95	-	-	79.0
		25 °C	5.0	4.15	-	-	82.8
Bi	250	60 °C	5.0	0.56	-	-	11.3
		25 °C	5.0	0.66	-	-	13.2
Sb	600	60 °C	5.0	1.17	-	-	23.4
		25 °C	5.0	1.04	-	-	20.8

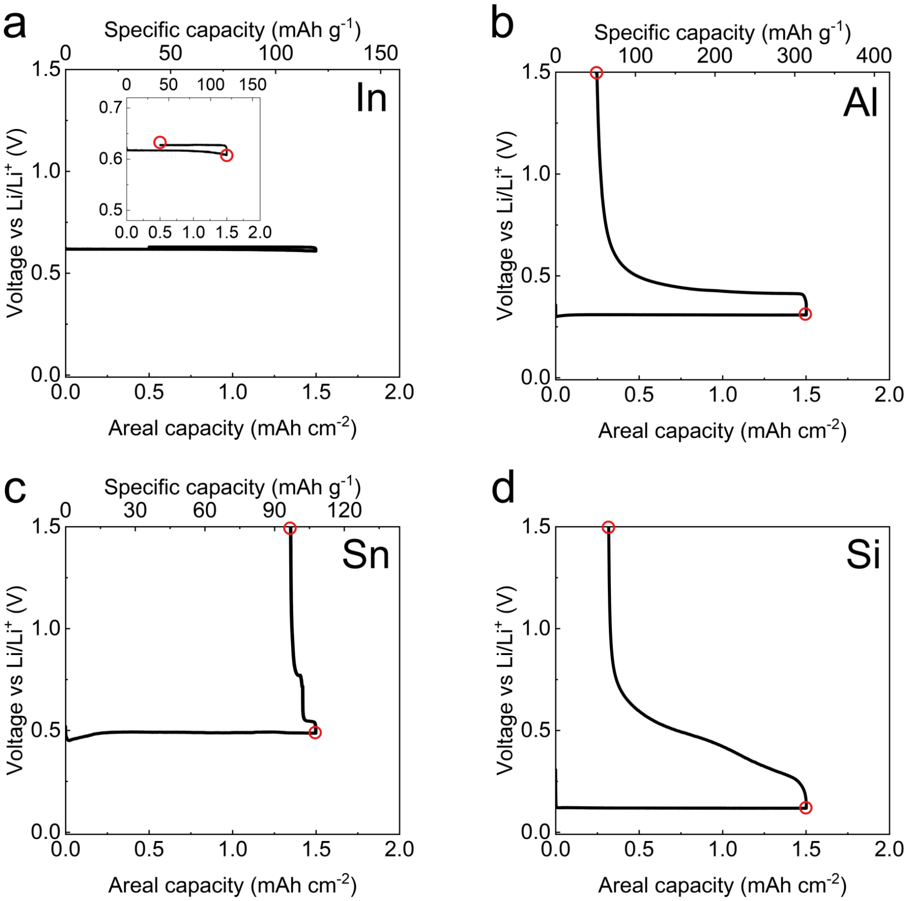

Figure S2. First-cycle galvanostatic voltage curves for cryo-FIB-SEM samples in Figure 2a-h. The solid-state half cells with indium, aluminum, tin, and silicon electrodes were lithiated to a capacity of 1.5 mAh cm^{-2}, which is a capacity value that is less than the full theoretical capacity of the electrodes. (a) Indium foil was partially delithiated to a capacity cutoff of 1.0 mAh cm^{-2}. The inset shows the voltage curve in the range of 0.5 to 0.7 V vs. Li/Li^{+}. (b-d) Aluminum, tin, and silicon electrodes were delithiated to 1.5 V. Red circles on the voltage curves indicate the stages at which *ex situ* cryo-FIB-SEM imaging was performed. Half cells were tested under a uniaxial stack pressure of 8 MPa and a current density of 50 μA cm^{-2} at 25 °C. The specific capacities of the lithiated foils were 117.8 mAh g^{-1} for indium, 314.2 mAh g^{-1} for aluminum, and 107.6 mAh g^{-1} for tin.

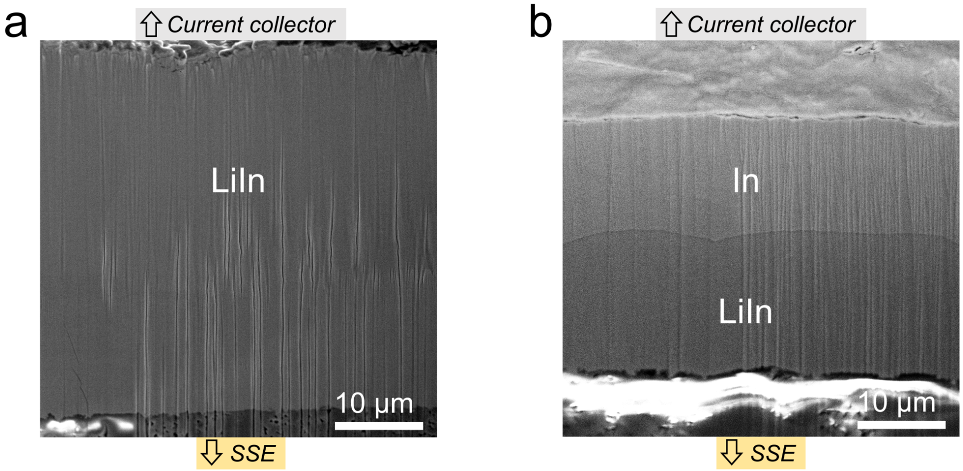

Figure S3. Cryo-FIB-SEM images of indium (a) after lithiation to 0.34 V vs. Li/Li^+ to completely react the foil to form the LiIn phase and (b) after delithiation to a capacity cutoff of 2.0 mAh cm^{-2}.

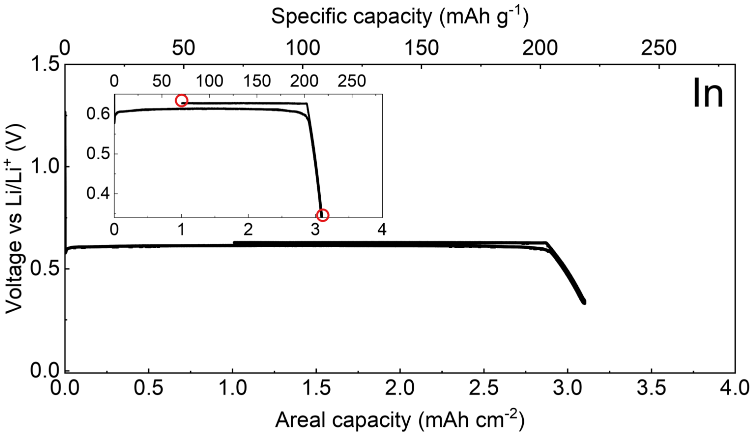

Figure S4. First-cycle galvanostatic voltage curve for cryo-FIB-SEM samples in Figure S2. The solid-state half cell with indium electrode was lithiated to 0.34 V vs. Li/Li^+ and delithiated to a capacity cutoff of 2.0 mAh cm^{-2}. The inset shows the voltage curve in the range of 0.34 to 0.65 V vs. Li/Li^+. Red circles on the voltage curve indicate the stages at which the *ex situ* cryo-FIB-SEM imaging was performed. The half cell was tested under a uniaxial stack pressure of 8 MPa and a current density of 50 μA cm^{-2} at 25 °C.

Table S3. Alloy anode thickness used in solid-state full cell tests, as well as first-cycle areal charge/discharge capacities, specific charge/discharge capacities, and Coulombic efficiency values from Figure 4.

Foil anode	Thickness (μm)	Areal charge capacity (mAh cm^{-2})	Areal discharge capacity (mAh cm^{-2})	Specific charge capacity (mAh g^{-1})	Specific discharge capacity (mAh g^{-1})	Specific charge capacity (mAh g^{-1}: NMC622)	Specific discharge capacity (mAh g^{-1}; NMC622)	Coulombic efficiency (%)
In	20	4.55	3.89	301.5	257.4	169.7	144.9	85.4
Al	20	5.00	3.45	975.6	672.5	185.8	128.1	68.9
Sn	10	5.00	2.51	680.6	340.3	185.8	92.9	50.0
Si	500	5.00	3.78	-	-	185.8	140.3	75.4

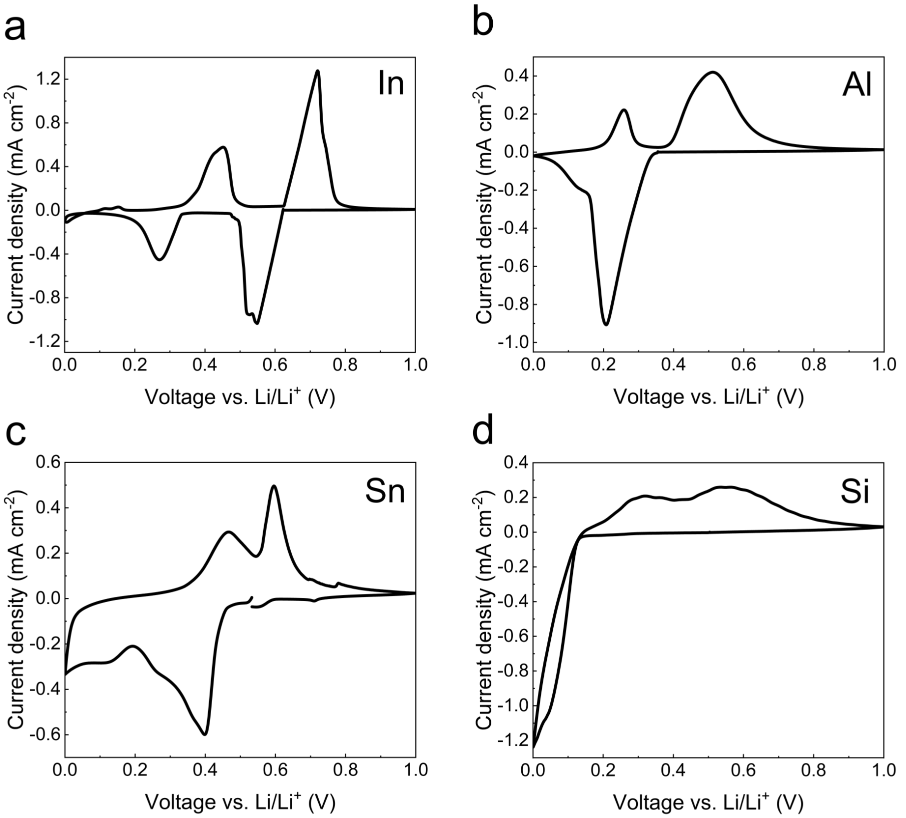

Figure S5. First-cycle cyclic voltammetry curves of alloy foil anodes in solid-state half cells utilizing LPSC SSE and lithium metal counter electrodes. (a) Indium, (b) aluminum, (c) tin, and (d) silicon. The sweep rate was 0.01 mV s^{-1} with a voltage range of 0 to 1.0 V. The half cells were tested at 25 °C with a uniaxial stack pressure of 8 MPa.

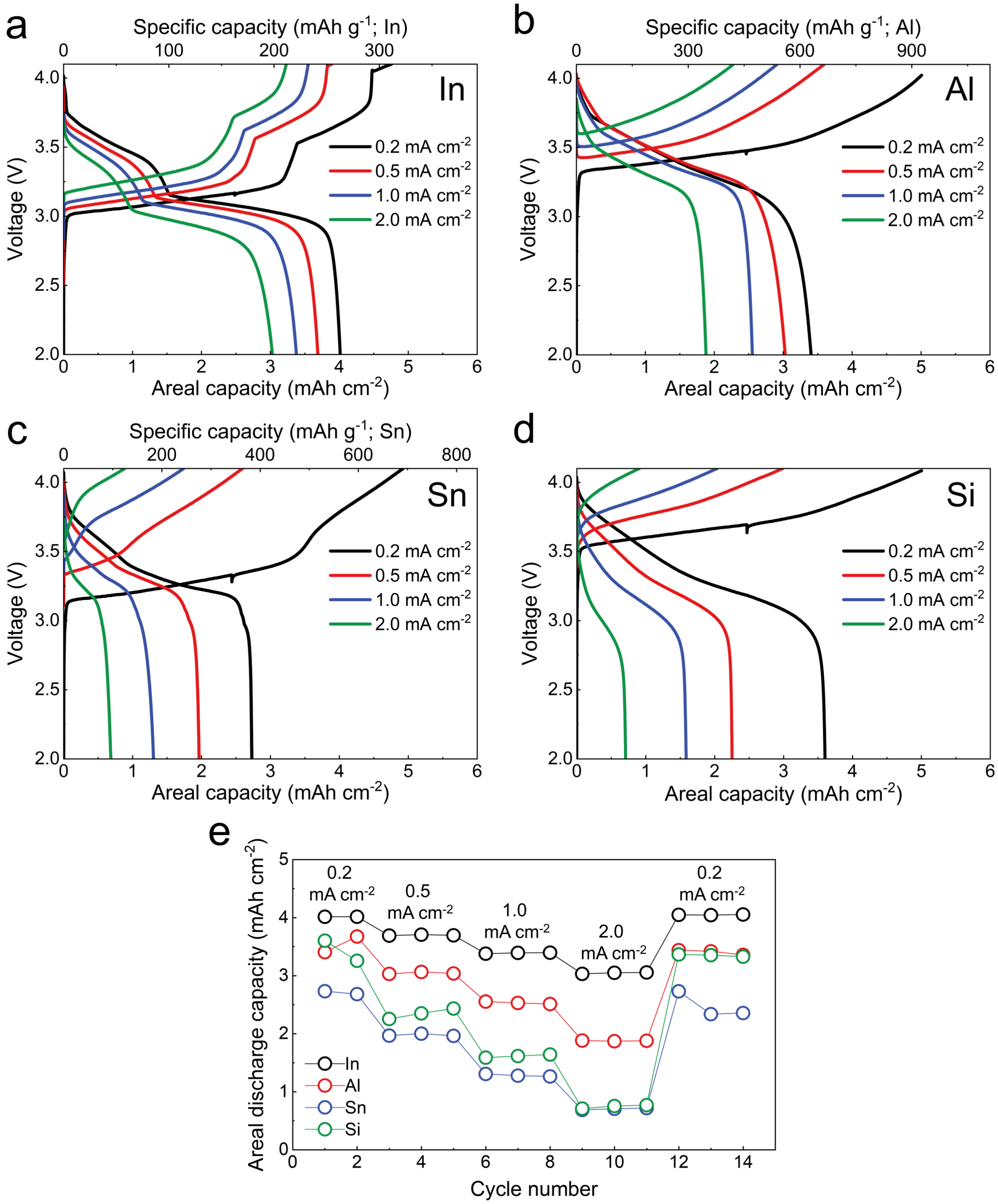

Figure S6. Rate-dependent galvanostatic testing of alloy foil anodes in solid-state full cells with LPSC SSE and NMC622 cathodes. (a-d) Voltage curves under different current densities from (a) indium (20 µm), (b) aluminum (20 µm), (c) tin (10 µm), and (d) silicon (500 µm) cells. The cathode loading and N:P ratios of these cells were set to be identical to those of the full cells tested in the galvanostatic cycling tests in Figure 4. (e) Discharge capacity values during this rate testing. The areal charge capacity was limited to 5 mAh cm^{-2} in each cycle. All cells were tested at 25 °C in the voltage range of 2.0 to 4.1 V with a uniaxial stack pressure of 8 MPa.

Supplemental References

(S1) Zhang, W.; Weber, D. A.; Weigand, H.; Arlt, T.; Manke, I.; Schröder, D.; Koerver, R.; Leichtweiss, T.; Hartmann, P.; Zeier, W. G. Interfacial processes and influence of composite cathode microstructure controlling the performance of all-solid-State lithium batteries. *ACS Appl. Mater. Interfaces* **2017**, *9* (21), 17835-17845.

(S2) Lewis, J. A.; Lee, C.; Liu, Y.; Han, S. Y.; Prakash, D.; Klein, E. J.; Lee, H.-W.; McDowell, M. T. Role of areal capacity in determining short circuiting of sulfide-based solid-state batteries. *ACS Appl. Mater. Interfaces* **2022**, *14* (3), 4051-4060.

(S3) Tan, D. H. S.; Meng, Y. S.; Jang, J. Scaling up high-energy-density sulfidic solid-state batteries: A lab-to-pilot perspective. *Joule* **2022**, *6* (8), 1755-1769.

(S4) Lee, J. Z.; Wynn, T. A.; Schroeder, M. A.; Alvarado, J.; Wang, X.; Xu, K.; Meng, Y. S. Cryogenic focused ion beam characterization of lithium metal anodes. *ACS Energy Lett.* **2019**, *4* (2), 489-493.
